# Catalytic reduction of 4-nitrophenol with gold nanoparticles synthesized by caffeic acid

**DOI:** 10.1186/s11671-016-1776-z

**Published:** 2017-01-05

**Authors:** Yu Seon Seo, Eun-Young Ahn, Jisu Park, Tae Yoon Kim, Jee Eun Hong, Kyeongsoon Kim, Yohan Park, Youmie Park

**Affiliations:** 1 College of Pharmacy and Inje Institute of Pharmaceutical Sciences and Research, Inje University, 197 Inje-ro, Gimhae, Gyeongnam 50834 Republic of Korea; 2 Department of Pharmaceutical Engineering, Inje University, 197 Inje-ro, Gimhae, Gyeongnam 50834 Republic of Korea

**Keywords:** Gold nanoparticles, Caffeic acid, Catalytic activity, 4-Nitrophenol reduction reaction, Centrifugation

## Abstract

In this study, various concentrations of caffeic acid (CA) were used to synthesize gold nanoparticles (CA-AuNPs) in order to evaluate their catalytic activity in the 4-nitrophenol reduction reaction. To facilitate catalytic activity, caffeic acid was removed by centrifugation after synthesizing CA-AuNPs. The catalytic activity of CA-AuNPs was compared with that of centrifuged CA-AuNPs (*cf*-CA-AuNPs). Notably, *cf*-CA-AuNPs exhibited up to 6.41-fold higher catalytic activity compared with CA-AuNPs. The catalytic activity was dependent on the caffeic acid concentration, and the lowest concentration (0.08 mM) produced CA-AuNPs with the highest catalytic activity. The catalytic activities of both CA-AuNPs and *cf*-CA-AuNPs decreased with increasing caffeic acid concentration. Furthermore, a conversion yield of 4-nitrophenol to 4-aminophenol in the reaction mixture was determined to be 99.8% using reverse-phase high-performance liquid chromatography. The product, 4-aminophenol, was purified from the reaction mixture, and its structure was confirmed by ^1^H-NMR. It can be concluded that the removal of the reducing agent, caffeic acid in the present study, significantly enhanced the catalytic activity of CA-AuNPs in the 4-nitrophenol reduction reaction.

## Background

For many years, gold had been considered as an inert metal. The first discovery of gold nanoparticles (AuNPs) in the catalytic field was an oxidation of carbon monoxide by AuNPs supported on the transition metal oxide [1]. Acting as catalysts in organic reactions, AuNPs have attracted considerable attention due to their unique physical and chemical properties [2–4]. One of its merits in catalysis is that many organic reactions can be achieved under mild conditions, and the high surface-area-to-volume ratio of AuNPs leads to increase in chemical reactivity [5]. Examples of organic reactions that use AuNPs include (i) hydrogenation reactions of unsaturated carbonyls and reduction of nitro groups, (ii) alkyne activation, (iii) coupling reactions, and (iv) oxidation reactions of cyclohexane, toluene, alcohols, and alkenes [5].

To assess the catalytic activity of AuNPs, the reduction reaction of 4-nitrophenol (4-NP) to 4-aminophenol (4-AP) with excess NaBH_4_ is generally employed as a model reaction [6]. 4-NP and its derivatives are used to manufacture herbicides, insecticides and synthetic dyestuffs, and they can substantially damage the ecosystem with common organic pollutants of wastewater [7, 8]. The reaction product, 4-AP, is a useful compound used as an intermediate for manufacturing analgesics and antipyretics.

Recently, many researchers have actively studied green synthetic methods using biological entities as reducing agents to convert Au ions to AuNPs. Such methods eliminate the use of toxic chemicals and increase the biocompatibility of the resulting AuNPs. Moreover, these methods also have the benefits of using aqueous solvents, conducting reactions in one-pot and being eco-friendly. In this study, caffeic acid, one of phenolic compounds in plants, was used as a reducing agent to synthesize AuNPs (referred to hereafter as CA-AuNPs). Caffeic acid is abundant in honey, olive oil, coffee beans and medicinal plants. Caffeic acid and its derivatives have a variety of biological activities, including anti-atherosclerotic, anti-bacterial, anti-cancer, anti-inflammatory, anti-oxidative, anti-viral, immunostimulatory, and neuroprotective properties [9–16].

There are research reports regarding the enhancement of catalytic activity of colloidal AuNPs [17–19]. Kim and coworkers designed anisotropic/partially aggregated AuNPs possessing a strong and wide absorbance in visible and near-infrared light to enhance reaction rates of 4-NP to 4-AP under light irradiation [17]. Upon light irradiation, the anisotropic/partially aggregated ones efficiently convert photon to heat, thus, the reaction rate of 4-NP to 4-AP increased notably, whereas the monodispersed ones showed only moderate increase in reaction rates [17]. Recently, You and coworkers have reported the surface modification of metallic nanoparticles by changing capping ligands for the enhancement of catalytic activity [18]. They modified the surface of metallic nanoparticles from citrate with a cationic polymer, poly(diallyldimethylammonium chloride) (PDDA) capping. The PDDA produced net positive charges on the surface of metallic nanoparticles which afforded strong electrostatic attraction between the surface and negatively charged ions (nitrophenolate and borohydride ions) and finally enhanced catalytic activity in the 4-NP reduction reaction [18]. Most recently, we green-synthesized AuNPs with *Artemisia capillaris* extract and their catalytic activity was assessed in the 4-NP reduction reaction [19]. The AuNPs were centrifuged and re-dispersed with water to remove extract on the surface and the catalytic activity of the initial AuNPs and the centrifuged AuNPs were compared. Remarkably, the centrifuged AuNPs exhibited an enhancement of catalytic activity up to 50.4% [19]. In addition to the size and shapes of metallic nanoparticles, the surface modification by removal of capping agents is another crucial factor to control catalytic activity. This can be explained by Langmuir-Hinshelwood mechanism in the following.

Langmuir-Hinshelwood mechanism was proposed by Wunder and co-workers as a mechanistic model for the reduction of 4-NP by NaBH_4_ in the presence of metallic nanoparticles [20, 21]. According to their proposed model, the surface of metallic nanoparticles serves as the location where the catalytic reduction process occurs. Borohydride ions bind to the surface, and concomitantly, 4-NP also adsorbs on the surface. Subsequently, 4-NP is reduced by borohydride ions to 4-AP, which is a rate-determining step. Ciganda and co-workers have reported “restructuration” on the surface during the catalytic process, where ligands are displaced by substrates on the surface [22]. Restructuration on the surface is a dominant aspect of the Langmuir-Hinshelwood mechanism. Generally, no induction time is observed when a very facile ligand displacement occurs by substrates. In contrast, stronger binding of ligands on the surface is responsible for their difficult displacement by substrates, leading to longer induction times. Based on previous reports [20–22], we hypothesized that removal of the ligand (caffeic acid in the present study) will considerably affect the “restructuration” process on the surface and facilitate the displacement process between ligands and substrates. This will lead to an increase in rate constants and finally enhance the catalytic activity.

Therefore, the present report focuses on (i) evaluating the catalytic activities of CA-AuNPs synthesized under various concentrations of caffeic acid, (ii) removing caffeic acid by centrifugation (referred to hereafter as *cf*-CA-AuNPs) and comparing the catalytic activity of CA-AuNPs with that of *cf*-CA-AuNPs, (iii) obtaining a conversion yield by reverse-phase high performance liquid chromatography (RP-HPLC), and finally (iv) purifying 4-AP by silica gel column chromatography and characterizing it by ^1^H-NMR. The reduction reaction of 4-NP to 4-AP in the presence of NaBH_4_ was selected as the model reaction.

## Methods

### Materials

Hydrochloroauric acid trihydrate (HAuCl_4_∙3H_2_O), caffeic acid, 4-nitrophenol, 4-aminophenol, ninhydrin, acetic acid and NaBH_4_ were purchased from Sigma-Aldrich (St. Louis, MO, USA). DMSO-d_6_ was obtained from Armar Chemicals (Dottingen, Switzerland). All other reagents were of analytical grade. TLC analyses were performed using Merck pre-coated TLC plates (silica gel 60 GF254, 0.25 mm, Germany). Syringe filtration was conducted using Minisart RC syringe filters (hydrophilic, 0.2 μm, Sartorius Stedim Biotech GmbH, Goettingen, Germany). All solutions were prepared in deionized water.

### Instruments

UV-visible spectra were acquired on a Shimadzu UV-2600 using a quartz cuvette (Shimadzu Corporation, Kyoto, Japan). Hydrodynamic size measurements by dynamic light scattering were performed using a NanoBrook 90Plus Zeta (Brookhaven Instruments Corporation, Holtsville, NY, USA). A JEOL JEM-3010 TEM operating at an accelerating voltage of 300 kV was used to acquire high-resolution transmission electron microscopy (HR-TEM) images (JEOL Ltd., Tokyo, Japan). The nanoparticle solution was pipetted onto a carbon-coated copper grid (carbon type B, 300 mesh, Ted Pella Inc., Redding, CA, USA), and the sample-loaded grid was dried for 12 h at room temperature prior to HR-TEM analysis. The crystalline nature of the AuNPs was analyzed using high-resolution X-ray diffraction (HR-XRD) with a Bruker D8 Discover high-resolution X-ray diffractometer in the range of 20° to 90° (2θ scale). HR-XRD was equipped with a Cu-Kα radiation source (*λ* = 0.154056 nm) (Bruker, Germany). The powdered sample was prepared using a FD8518 freeze-dryer (IlShinBioBase Co. Ltd., Gyeonggi-do, Republic of Korea). A Varian 500 MHz spectrometer was used to acquire ^1^H-NMR spectra (Palo Alto, CA, USA).

### Preparation of CA-AuNPs and *cf*-CA-AuNPs

**Fig. 1:**
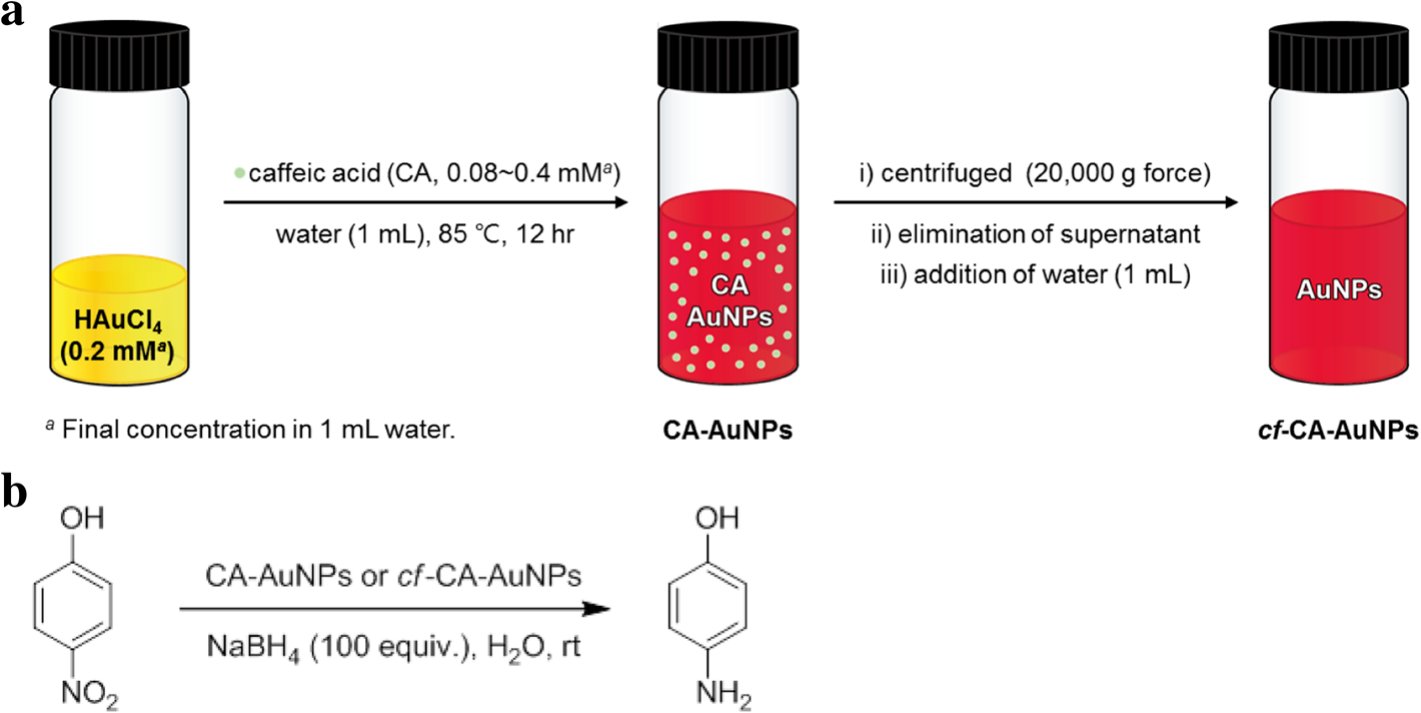
Schematic representation of **a** preparation of AuNPs catalyst with different concentrations of caffeic acid, and **b** reduction reaction of 4-NP to 4-AP with CA-AuNPs or *cf*-CA-AuNPs catalyst

A schematic representation for preparing both CA-AuNPs and *cf*-CA-AuNPs is presented in Fig. 1a. CA-AuNPs were synthesized using various concentrations of caffeic acid according to our previous report [23]. Final concentrations of caffeic acid for the synthesis of CA-AuNPs in the present study were 0.08, 0.12, 0.16, 0.20, 0.24, 0.28, 0.32, 0.36, and 0.40 mM under a fixed final concentration of hydrochloroauric acid trihydrate (0.2 mM) in a final volume of 1 mL. An incubation was performed in an 85 °C dry oven for 12 hrs. *cf*-CA-AuNPs were prepared by centrifugation (20,000*g* force, 24 °C, 30 min) using a 5424R centrifuge (Eppendorf AG, Hamburg, Germany). After centrifugation, the supernatant containing caffeic acid was removed, and deionized water was added to a pellet to make a final volume of 1 mL. The centrifuged and re-dispersed solution was labeled as *cf*-CA-AuNPs.

### Catalytic activity in the 4-NP reduction reaction

A schematic representation of the 4-NP reduction reaction is presented in Fig. 1b. 4-NP (0.4 mM, 1 mL) was mixed with deionized water (1.8 mL) in a quartz cuvette, then added with an aqueous solution of NaBH_4_ (200 mM, 200 μL). To this mixture, either CA-AuNPs (1 mL) or *cf*-CA-AuNPs (1 mL) was added as a catalyst. Final concentrations of 4-NP, NaBH_4_, and Au atoms in 4 mL water were 0.1, 10, and 0.05 mM, respectively. The molarity of Au atoms was calculated based on a final concentration of hydrochloroauric acid trihydrate (0.2 mM). The reaction progress was monitored using a UV-visible spectrophotometer.

### Calculation of conversion yield in the 4-NP reduction reaction

A conversion yield from 4-NP to 4-AP was determined using RP-HPLC. Shimadzu RP-HPLC system (CBM-20A) was composed of an autosampler (SIL-20 AC), a pump (LC-20AT), a column oven (CTO-20A), a UV detector (SPD-M20A), and a degassing unit (DGU-20A_5R_). A Thermo AQUASIL C18 column (150 mm length × 4.6 mm i.d., 5 μm particle size) was used with an isocratic elution. Mobile phase was consisted of 10% acetonitrile in ammonium bicarbonate buffer (10 mM, pH 8.11). The injection volume was 10 μL with a flow rate of 0.8 mL/min. Column oven temperature was set at 30 °C, and UV detection wavelength was at 254 nm. The 4-AP standard was dissolved in deionized water to produce a standard stock solution (50 mM). A calibration curve was established based on six concentrations of 4-AP standard solutions in the range of 0.0125 to 0.2 mM by serial dilution of the standard stock solution with deionized water. A linearity was observed in a concentration range of 0.0125 to 0.2 mM with a regression equation of *y* = 840160 × + 14095 (*r* ^2^ = 0.999). After completion of the reduction reaction of 4-NP to 4-AP by *cf*-CA-AuNPs, the reaction mixture was filtered using Minisart RC syringe filters (hydrophilic, 0.2 μm) prior to RP-HPLC injection. To calculate the conversion yield, *cf*-CA-AuNPs that were synthesized using the lowest caffeic acid concentration (0.08 mM) were employed as a catalyst.

### Purification and characterization of 4-AP

The mixture was prepared by mixing 4-NP (10 mM, 4.5 mL), an aqueous solution of NaBH_4_ (300 mM, 7.5 mL) and deionized water (8 mL). To this reaction mixture, CA-AuNPs synthesized with 0.08 mM caffeic acid (2.5 mL) were added, and the mixture was stirred for 20 min. The final concentrations of reagents in deionized water (22.5 mL) were as follows: 4-NP (2 mM), NaBH_4_ (100 mM), Au atoms (0.022 mM), and caffeic acid (3.56 μM). After completion of the reaction, HCl solution (2 M, 3 mL) was added to quench the reaction. Then, the reaction mixture was neutralized by adding NaOH (1 M, 4.5 mL). To this solution, ethyl ether (30 mL) was added, and a liquid-liquid extraction was conducted. The extraction step was repeated three times. Ethyl ether fractions containing 4-AP were pooled, and moisture was removed using Na_2_SO_4_. Then, ethyl ether was evaporated under reduced pressure. A silica gel column chromatography (6.5 cm diameter × 23 cm length) was performed by eluting with solvents composed of hexane:ethyl acetate (2:1→1:2). Each eluent was loaded on a TLC plate together with 4-AP standard and developed with a ninhydrin reagent. The fractions containing 4-AP were pooled, and solvents were evaporated under reduced pressure. Both authentic 4-AP from a commercial source and purified 4-AP from the above procedure were characterized by ^1^H-NMR. Authentic 4-AP: ^1^H-NMR (500 MHz, DMSO-d_6_) δ 8.34 (s, 1H), 6.47–6.40 (dd, 4H, *J*_1_ = 30.5 Hz, *J*_2_ = 8.5 Hz), 4.37 (s, 2H); Purified 4-AP: ^1^H-NMR (500 MHz, DMSO-d_6_) δ 8.34 (s, 1H), 6.47–6.34 (dd, 4H, *J*_1_ = 30.5 Hz, *J*_2_ = 8.5 Hz), 4.42 (s, 2H).

## Results and discussion

### Green synthesis of CA-AuNPs with various concentrations of caffeic acid

**Fig. 2:**
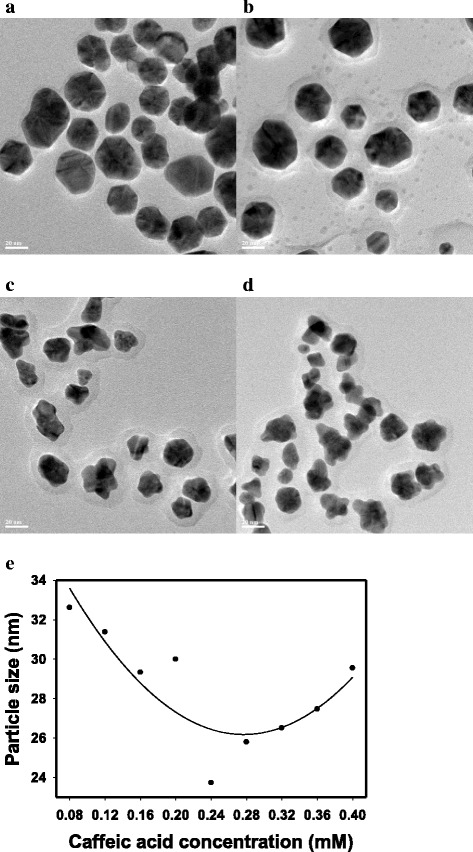
HR-TEM images of CA-AuNPs. Caffeic acid concentrations of **a** 0.08 mM, **b** 0.16 mm, **c** 0.32 mM and **d** 0.36 mM. The *scale bar* represents 20 nm in all images. **e** The relationship between the particle size (nm) measured from HR-TEM images and caffeic acid concentration (mM)

In our previous report, CA-AuNPs were synthesized using various concentrations of caffeic acid (0.008–0.56 mM) [23]. In this study, CA-AuNPs were synthesized using nine concentrations of caffeic acid in the range of 0.08 to 0.40 mM according to our previous report [23]. As summarized in Table 1, as caffeic acid concentrations increased, the surface plasmon resonance (SPR) bands of CA-AuNPs had a tendency to red-shift (537.5 to 587.0 nm). HR-TEM images of CA-AuNPs synthesized using four different concentrations of caffeic acid (0.08, 0.16, 0.32 and 0.36 mM) are shown in Fig. 2a–d. HR-TEM images of CA-AuNPs synthesized using the other concentrations are clearly presented in our previous report [23]. Shapes of CA-AuNPs changed from spherical to sea-urchin-like as the caffeic acid concentrations increased [23]. Approximately 100 discrete nanoparticles from each concentration were randomly selected from HR-TEM images to measure an average size. The average size was in the range of 23.73 to 32.61 nm. The smallest size (23.73 ± 3.20 nm) was obtained at a caffeic acid concentration of 0.24 mM, and a parabolic shape of particle size was observed in the plot (Fig. 2e). The size determined from HR-TEM images was in good agreement with the hydrodynamic size measurement results in our previous report [23]. “….the plot of the mean particle size exhibited a parabolic shape, where 0.28 mM caffeic acid resulted in the smallest particle size of 89.4 nm. The mean particle size increased when the concentration of caffeic acid was either higher or lower than 0.28 mM” [23]. The only difference is that the hydrodynamic size was affected by a solvent medium, particularly water; thus, the hydrodynamic size was larger than the size measured directly from HR-TEM images. The larger particle size from the hydrodynamic measurement is potentially due to a formation of hydration layers with caffeic acid on the surface of AuNPs. CA-AuNPs was acidic in the pH range of 3.92 to 4.36.

**Table 1:** CA-AuNPs and *cf*-CA-AuNPs synthesized using various concentrations of caffeic acid

Caffeic acid final concentrations		0.08 mM	0.12 mM	0.16 mM	0.20 mM	0.24 mM	0.28 mM	0.32 mM	0.36 mM	0.40 mM
CA-AuNPs	Absorbance	0.541	0.823	1.012	1.101	1.130	1.045	1.056	1.066	1.059
	Wavelength (nm)	537.5	549.0	545.0	535.0^a^	544.0	550.5	561.5	576.5	587.0
	Particle size (nm) from HR-TEM images	32.61 ± 6.13	31.37 ± 6.06	29.33 ± 5.41	29.99 ± 7.43^b^	23.73 ± 3.20	25.79 ± 4.73	26.51 ± 5.27	27.46 ± 5.82	29.55 ± 7.43
	Number of particles in HR-TEM taken for size measurement	132	88	137	123^c^	148	128	125	104	105
	Hydrodynamic size (nm)^d^	619.6	423.1	153.4	109.6	95.4	89.4	-	-	222.6
	pH	4.36	4.29	4.23	4.09	4.07	4.15	4.11	3.92	3.87
*cf*-CA-AuNPs	Absorbance	0.455	0.677	0.724	0.812	0.896	0.680	0.705	0.769	0.722
	Wavelength (nm)	538.0	554.0	550.50	548.5	547.0	555.5	568.0	584.0	592.5
	Hydrodynamic size (nm)	155.3	134.0	104.4	80.6	72.0	75.4	88.6	96.2	122.6
	pH	5.18	5.47	5.41	5.22	5.17	5.20	5.04	5.30	5.18
Absorbance ratio [*cf*-CA-AuNPs/CA-AuNPs] × 100 (%)		84.1	82.3	71.5	73.8	79.3	65.1	66.8	72.1	68.2

^a,b,c^These values were taken from our previous report [30].

^d^Hydrodynamic size (nm) was taken from our previous report [23].

### Centrifugation of CA-AuNPs to generate *cf*-CA-AuNPs

**Fig. 3:**
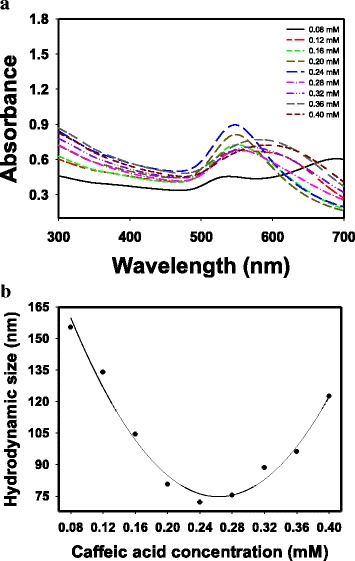
**a** UV-visible spectra of *cf*-CA-AuNPs, and **b** the relationship between hydrodynamic size (nm) and caffeic acid concentration (mM)

To remove caffeic acid from CA-AuNPs, centrifugation was performed. After centrifugation, a supernatant containing caffeic acid was removed. Then, a pellet was re-dispersed with the same volume of deionized water, and the re-dispersed solution was labeled as *cf*-CA-AuNPs. UV-visible spectra of *cf*-CA-AuNPs are shown in Fig. 3a. As caffeic acid concentrations increased, UV-visible spectra became broader with a red-shift. The centrifugation process resulted in a decreased absorbance in *cf*-CA-AuNPs (Table 1). The absorbance of *cf*-CA-AuNPs was in the range of 65.1 to 84.1% when the absorbance of CA-AuNPs was set at 100%. The hydrodynamic size (72.0–155.3 nm) was smaller than that of CA-AuNPs, suggesting that caffeic acids on the surface of AuNPs were successfully removed (Table 1). Additionally, as shown in Fig. 3b, a parabolic shape was also observed in the plot of *cf*-CA-AuNPs. The caffeic acid concentration of 0.24 mM resulted in the smallest hydrodynamic size of 72 nm. *cf*-CA-AuNPs was acidic in the pH range of 5.04 to 5.47, which was one pH unit higher compared with that of CA-AuNPs. This result also indicated that caffeic acids were successfully removed by centrifugation, which resulted in an increase of one pH unit.

### Catalytic activity in the 4-NP reduction reaction

**Fig. 4:**
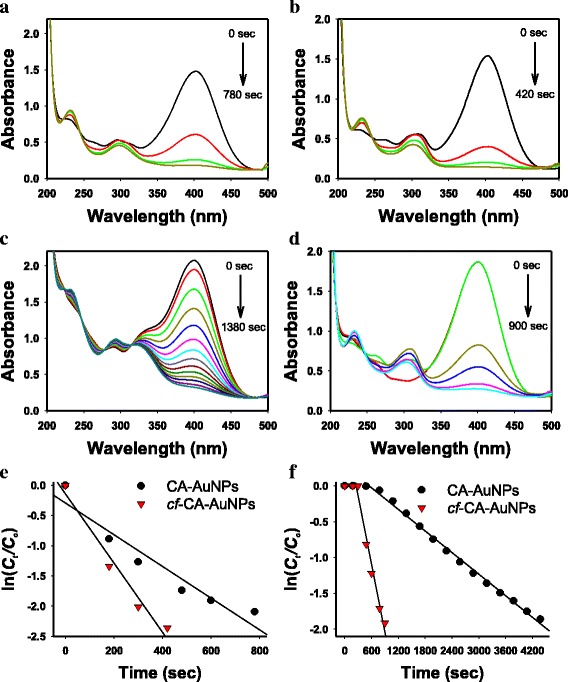
**a**–**d** The 4-NP reduction reaction monitored by UV-visible spectrophotometry. **a** CA-AuNPs (0.08 mM), **b** *cf*-CA-AuNPs (0.08 mM), **c** CA-AuNPs (0.32 mM), and **d** *cf*-CA-AuNPs (0.32 mM). **e**, **f** The plot of ln(*C*_*t*_ */C*_*0*_) vs time (sec). **e** caffeic acid concentration of 0.08 mM and **f** caffeic acid concentration of 0.32 mM

**Fig. 5:**
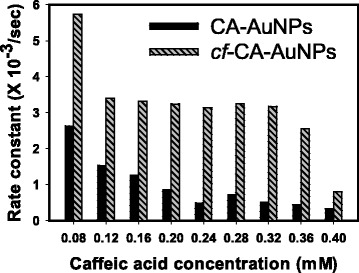
Rate constants for the 4-NP reduction reaction with CA-AuNPs or *cf*-CA-AuNPs

The 4-NP reduction reaction is generally selected as a model reaction for evaluating the catalytic activity of AuNPs. Many research articles have presented the catalytic activities of green-synthesized AuNPs using the 4-NP reduction reaction [24–29]. A primary reason for selecting the 4-NP reduction reaction is that a reaction progress is easily monitored by using UV-visible spectrophotometry. When 4-NP was mixed with NaBH_4_, the solution became yellow and had a maximum absorbance at 400 nm due to the formation of 4-nitrophenolate anions. To ensure pseudo-first-order kinetics, an excess amount (100-fold excess in the present study) of NaBH_4_ relative to 4-NP concentration was utilized. Without the addition of AuNPs as a catalyst, the peak of 4-nitrophenolate anion at 400 nm did not change. Upon the addition of either CA-AuNPs or *cf*-CA-AuNPs, the peak at 400 nm started to decrease while a new peak at 300 nm simultaneously appeared (Fig. 4a–d). The new peak at 300 nm exhibited an absorbance corresponding to a reaction product 4-AP. A reaction rate was measured from the plot between ln(*C*_*t*_ */C*_*0*_) and time (sec). Herein, the 4-NP concentration at time *0* and time *t* were expressed as *C*_*0*_ and *C*_*t*_, respectively. A linear relationship between ln(*C*_*t*_ */C*_*0*_) and time (sec) was shown, suggesting that the reaction followed pseudo-first-order kinetics (Fig. 4e,f). Rate constants are presented in Table 2, Figs. 4 and 5.

**Table 2:** Induction time and rate constant of the 4-NP reduction reaction in the presence of either CA-AuNPs or *cf*-CA-AuNPs

Caffeic acid final concentrations		0.08 mM	0.12 mM	0.16 mM	0.20 mM	0.24 mM	0.28 mM	0.32 mM	0.36 mM	0.40 mM
Induction time (s)	CA-AuNPs	0	0	0	0	180	480	780	1080	1380
	*cf*-CA-AuNPs	0	0	0	0	0	0	300	300	780
Rate constant (s^−1^)	CA-AuNPs	2.63 × 10^-3^	1.53 × 10^-3^	1.26 × 10^-3^	0.86 × 10^-3a^	0.49 × 10^-3^	0.72 × 10^-3^	0.51 × 10^-3^	0.44 × 10^-3^	0.33 × 10^-3^
	*cf*-CA-AuNPs	5.73 × 10^-3^	3.40 × 10^-3^	3.32 × 10^-3^	3.24 × 10^-3^	3.14 × 10^-3^	3.25 × 10^-3^	3.17 × 10^-3^	2.55 × 10^-3^	0.80 × 10^-3^
	Fold of increase	2.18	2.22	2.63	3.77	6.41	4.51	6.21	5.80	2.42

^a^This value was taken from our previous report [30]

Rate constants revealed that the catalytic activity of *cf*-CA-AuNPs was higher than that of CA-AuNPs (Table 2 and Fig. 5). The rate constants of CA-AuNPs were in the range of 0.33 × 10^-3^ to 2.63 × 10^-3^∙s^-1^, and there was a decreasing tendency with increasing caffeic acid concentration. The same tendency was also observed in case of *cf*-CA-AuNPs (0.80 × 10^−3^–5.73 × 10^−3^∙s^−1^). According to these results, removing caffeic acid by centrifugation enhanced the catalytic activities by 2.18–6.41-fold. Interestingly, the rate constant was the highest when the lowest caffeic acid concentration (0.08 mM) was used for the synthesis, i.e., 2.63 × 10^−3^∙s^−1^ for CA-AuNPs and 5.73 × 10^−3^∙s^−1^ for *cf*-CA-AuNPs.

As mentioned previously, the induction time was affected by displacement of ligands with substrates on the surface of metallic nanoparticles. As expected, with increasing caffeic acid concentrations, longer induction times were observed for both CA-AuNPs and *cf*-CA-AuNPs (Table 2). This is because more time was required to displace a higher concentration of caffeic acid with 4-NP on the surface. No induction time (0 s) was observed at low concentrations: 0.08–0.20 mM for CA-AuNPs and 0.08–0.28 mM for *cf*-CA-AuNPs. At high concentrations, the induction time increased from 180 s (at 0.24 mM) to 1380 s (0.40 mM) for CA-AuNPs. In case of *cf*-CA-AuNPs, the induction time was remarkably shorter than that of CA-AuNPs. Consequently, the removal of caffeic acid by centrifugation resulted in a fast restructuration on the surface and reduced the induction time, which directly increased the rate constant.

Why did the lowest concentration of caffeic acid show the highest rate constant for both CA-AuNPs and *cf*-CA-AuNPs? For CA-AuNPs, the rate constant gradually decreased with increasing caffeic acid concentration (Fig. 5). Thus, we are able to state that a major factor affecting the catalytic activity of CA-AuNPs was the caffeic acid concentration. In case of *cf*-CA-AuNPs, the lowest concentration (0.08 mM) showed the highest rate constant. For intermediate concentrations (0.12–0.32 mM), the rate constants remained almost constant without significant changes. The rate constant began to decrease at 0.32 mM, and the lowest rate constant was found at 0.40 mM. We assumed that caffeic acid was completely removed after centrifugation; thus, the major factor affecting the catalytic activity of *cf*-CA-AuNPs was most likely the shapes. With the lowest concentration of 0.08 mM, the shapes were spherical and amorphous, and it exhibited the highest rate constant (5.73 × 10^−3^∙s^−1^). Regardless of size, the rate constants of spherical shapes were nearly constant (3.17 × 10^−3^∙s^-1^–3.40 × 10^−3^∙s^−1^). The lowest rate constant was found in sea-urchin-like shapes. The larger the size in the sea-urchin-like shape was, the lower the catalytic activity was; i.e. 0.32 mM (3.17 × 10^−3^∙s^−1^, induction time of 300 s), 0.36 mM (2.55 × 10^−3^∙s^−1^, induction time of 300 s) and 0.40 mM (0.80 × 10^−3^∙s^−1^, induction time of 780 s). Furthermore, the longer induction time was observed with larger sea-urchin-like shapes.

### HR-XRD of CA-AuNPs (0.08 mM)

**Fig. 6:**
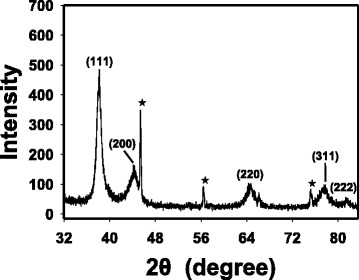
HR-XRD analysis of CA-AuNPs synthesized using a caffeic acid concentration of 0.08 mM

As we reported previously, CA-AuNPs synthesized using a caffeic acid concentration of 0.20 mM possessed a face-centered cubic structure [30]. In this study, CA-AuNPs that were synthesized using a caffeic acid concentration of 0.08 mM exhibited the best activity among the tested concentrations; thus, crystalline nature was again confirmed by HR-XRD pattern (Fig. 6). Strong diffraction peaks at 38.20°, 44.34°, 64.74°, 77.56°, and 81.36° corresponded to the (111), (200), (220), (311), and (222) planes of the face-centered cubic structure of crystalline Au.

### Conversion yield and purification/characterization of 4-AP

**Fig. 7:**
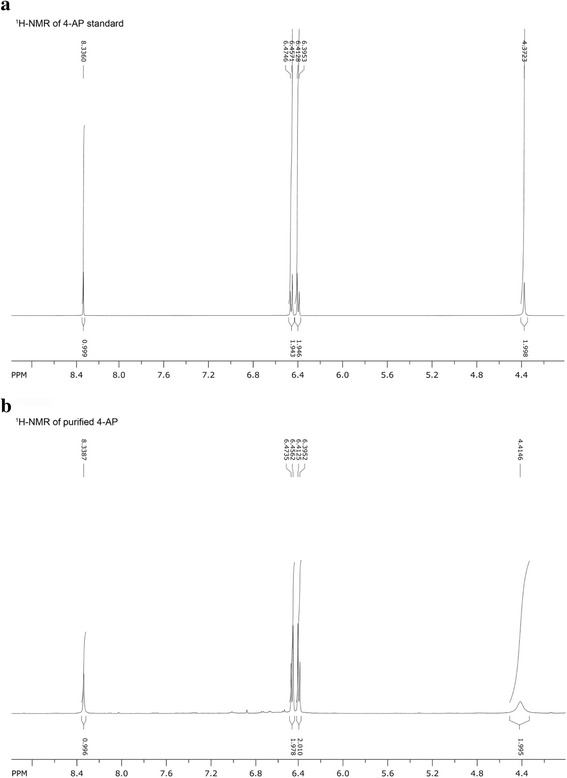
^1^H-NMR spectra of **a** authentic 4-AP and **b** purified 4-AP

To calculate a conversion yield, the reaction mixture was analyzed using RP-HPLC. In RP-HPLC chromatograms, a retention times for 4-AP and 4-NP were 4.0 and 4.6 min, respectively (data not shown). For a quantitative analysis, we constructed a calibration curve with 4-AP standard solutions and observed a linear regression equation of *y* = 840160 × + 14095 with a correlation coefficient of 0.999 in the concentration range of 0.0125–0.2 mM (data not shown). Based on this equation, the conversion yield of 4-NP to 4-AP was measured to be 99.8%. For the purification of 4-AP, we scaled up the reaction with 4-NP (2 mM), NaBH_4_ (100 mM), and Au atoms (0.022 mM) in 22.5 mL of water. After the work-up procedures, 4-AP was purified by silica gel column chromatography and its structure was determined by ^1^H-NMR. In comparison with the authentic 4-AP (Fig. 7a), the purified 4-AP (Fig. 7b) was confirmed to be the same compound.

### Comparison of catalytic activities of green-synthesized AuNPs in 4-NP reduction reaction

Comparisons of the catalytic activities of *cf*-CA-AuNPs in 4-NP reduction reaction with the green-synthesized AuNPs are shown in Table 3. Additionally, the amounts of substrates (4-NP), reductants (NaBH_4_), and catalysts (Au atoms) in each reduction of 4-NP were carefully examined. *cf*-CA-AuNPs (entry 1) possessed the largest size (38.61 ± 6.21 nm) among the green-synthesized AuNPs. All shapes of AuNPs were spherical, except for AuNPs synthesized with catechin (entry 6). In the present study, 4-NP reduction reaction by *cf*-CA-AuNPs shows the highest rate constant among entries 1–7, where 100 equivalents of NaBH_4_ and 0.5 equivalents of Au atoms per 4-NP were used (entry 1). In entries 2, 3 and 5, although less than 0.5 equivalents of Au atoms (0.2–0.42 equivalents) were used, additional 20–200 equivalents of NaBH_4_ (120 and 300 equivalents) were necessary to complete the reduction reaction. However, in case of entries 6 and 7, the same equivalents of NaBH_4_ (100 equivalents) were utilized, and the equivalents of Au atoms were 2.8–4.4-fold higher than entry 1. In case of entry 4, both equivalents of NaBH_4_ and Au atoms were 1.9- and 5.2-fold higher than entry 1, respectively. Thus, the relatively low equivalents of NaBH_4_ (100 equiv.) and Au atoms (0.5 equiv.) and high rate constant (5.73 × 10^−3^∙s^−1^) support that *cf*-CA-AuNPs are economical and effective green-synthesized AuNPs for 4-NP reduction reaction.

**Table 3:** Comparison of the catalytic activities of green-synthesized AuNPs in the 4-NP reduction reaction

Entry	Reducing agent used for synthesis of AuNPs	Particle size (nm)	Shape	Rate constant (s^-1^)	4-NP (mM)^*a*^	NaBH_4_ (mM)^*a*^	Au atoms (mM)^*a,b*^	Refs
1	Caffeic acid (*cf*-CA-AuNPs)	38.61 ± 6.21	Spherical	5.73 × 10^-3^	0.1	10 (100 equiv.)	0.05 (0.5 equiv.)	In the present study
2	*Punica granatum* juice	23.1–35.8	Spherical	3.67 × 10^-3^	0.05	15 (300 equiv.)	0.0135 (0.27 equiv.)	[24]
3	*Saraca indica* bark extract	17.97	Spherical	4.83 × 10^-3^	0.05	15 (300 equiv.)	0.021 (0.42 equiv.)	[25]
4	*Aerva lanata* leaf extract	18.62	Spherical	2.78 × 10^-3^	0.053	10 (189 equiv.)	0.1383 (2.61 equiv.)	[26]
5	*Prunus domestica* extract	20 ± 6	Spherical	1.90 × 10^-3^	0.094	11.268 (120 equiv.)	0.019 (0.2 equiv.)	[27]
6	catechin	16.6	Diverse shapes	1.52 × 10^-3^	0.034	2.269 (67 equiv.)	0.075 (2.22 equiv.)	[28]
7	*Bupleurum falcatum* root extract	10.5 ± 2.3	Spherical	0.82 × 10^-3^	0.07	7 (100 equiv.)	0.1 (1.43 equiv.)	[29]

^a^Molarity of the final volume of solutions

^b^Calculated by initial molarity of HAuCl_4_∙3H_2_O to synthesize AuNPs

## Conclusions

In conclusion, the removal of caffeic acid by simple centrifugation significantly enhanced the catalytic activity of CA-AuNPs in 4-NP reduction reaction by up to 6.41-fold. The removal of reducing agents facilitates a fast restructuration process on the surface, reduces induction times, and increases rate constants, enhancing the catalytic activity. In this respect, the current approach can be applied or extended to other metallic nanoparticles to enhance their catalytic activity. Furthermore, we purified 4-AP and confirmed its structure by ^1^H-NMR. The conversion yield from 4-NP to 4-AP was measured by RP-HPLC with an excellent yield of 99.8%.
